# Introducing the Attitudes toward Technology Longitudinal Aging Study (ATLAS): Initial Wave 1 Findings

**DOI:** 10.1093/geroni/igab046.3410

**Published:** 2021-12-17

**Authors:** Robin Stuart

**Affiliations:** Florida State University, Tallahassee, Florida, United States

## Abstract

Differences between younger and older adults' use and adoption of technology have declined over the past two decades, though the mechanisms behind observed trends are uncertain. Few longitudinal studies have tried to capture detailed changes in technology attitudes, adoption, and usage over time among older adults. This presentation presents newly collected data from the first wave of the Attitudes toward Technology Longitudinal Aging Study (ATLAS), a 5-wave questionnaire-based longitudinal study of older adults' attitudes toward technology and levels of technology use (N = 88; Men = 30; Women = 58; Mage = 69.7 years). We present baseline characteristics of Wave 1 and explore predictors of technology use, adoption, and proficiency. Waves 2 through 5 will assess changes in these domains. Wave 1 results replicated previous findings in that older age was associated with lower computer and mobile device proficiency (computer: r = -.219*, p < .05 , mobile device: r = -.291**, p < .01). However, there was variability among both types of proficiency (McomputerProf = 27.39, SD = 3.57 ; MmobileProf = 31.52, SD = 9.21), indicating room for change over time. Both types of proficiency were correlated with level of technology use (computer: r = -.219*, p < .05 , mobile device: r = -.572***, p < .001). Taken together, these initial relationships suggest the possibility that future waves will see changes in technology use predicted by changes in age-related differences in technology proficiency and attitudes.

